# Acquired uterine arteriovenous malformation—Does it really exist? A case report

**DOI:** 10.1016/j.radcr.2023.08.035

**Published:** 2023-08-26

**Authors:** Josefina Larcombe, Andrea Stuart

**Affiliations:** a Department of Obstetrics and Gynaecology, Helsingborg Hospital, Helsingborg, Sweden; b Institute of Clinical Sciences, Dept of Obstetrics and Gynecology, Lund University, Lund, Sweden

**Keywords:** Arterial venous malformation, Ultrasound velocity, MRI, Hysterectomy, Invasive placenta disorder

## Abstract

Acquired arteriovenous malformations (AVM) of the uterus can cause life-threatening vaginal bleeding and are associated with previous pregnancy, abortion or pelvic trauma. The pathophysiology is not well understood and the diagnosis is usually made by greyscale ultrasound often with nonspecific imaging findings, hence making it difficult to establish a correct diagnosis and therefore also the true incidence. However, case reports have previously described a connection between AVM formation and placental invasive disorders. In this report we demonstrate a case of a woman diagnosed with an AVM by ultrasound, presenting with menorrhagia after a termination of pregnancy, resulting in an emergency hysterectomy where subsequently a vascular malformation was found in conjunction with a remnant of a placenta increta and a placental site nodule. We hence suggest the hypothesis that these conditions are part of the same pathological process in the spectrum of abnormal invasive placental disorders, and that in the setting of previous trophoblastic processes, vascular malformations may mimic AVMs and ought not in fact to be considered as true AVMs.

## Introduction

Arteriovenous malformations (AVM) are abnormal connections between high pressure arteries and low pressure veins that bypass the capillary system [1], and are caused by a triggering event that damage the uterine tissue, such as pregnancy, abortion, cesarean delivery, or pelvic trauma [1,2]

The treatment of AVMs depends on several factors, namely how hemodynamically stability and fertility wishes. Short-term management involves blood transfusions, applying a Foley catheter for tamponade in the uterine cavity, and the use of medical treatments such as 15-methyl-prostaglandin F2 alpha, methylergonovine or danazol [2]. These treatments are directed at controlling the acute bleeding. The usual clinical recommendation is expectant management or the use of medical therapies such as the combined oral contraception pill.

Transcatheter embolization of the uterine artery is a less invasive alternative to hysterectomy and allows for the possibility of preserving fertility.

We demonstrate a case of a woman presenting with menorrhagia after a termination of pregnancy, and following grayscale and Doppler ultrasound imaging diagnosed with an AVM which was initially treated conservatively. Due to intermittent massive hemorrhage, an emergency hysterectomy was performed, and the final histological diagnosis gave 2 potential conclusions to the diagnosis.

## Case report

A 32 year old white female, gravida 6 para 1, with a pregnancy was dated to 16 + 1 gestational weeks, and she subsequently underwent a termination of pregnancy using mifepristone and misoprostol treatment. Total bleeding was estimated to approximately 1000 mL and the patient was later discharged.

The patient presents to the gynecological emergency unit 6 weeks later with lengthy, recurrent and profuse vaginal bleeding. Gynecological examination was essentially unremarkable. A vaginal ultrasound showed heterogeneous tissue between the endometrium, measuring 17 mm. No blood flow was demonstrated around the tissue, but it was noted that the anterior wall of the uterus was highly vascularized. Laboratory test showed negative pregnancy test, hemoglobin of 109 g/L and C-reactive protein of <5. The patient was prescribed a course of medroxyprogesteronacetate during 10 days and then the contraceptive pill.

**Fig. 1:**
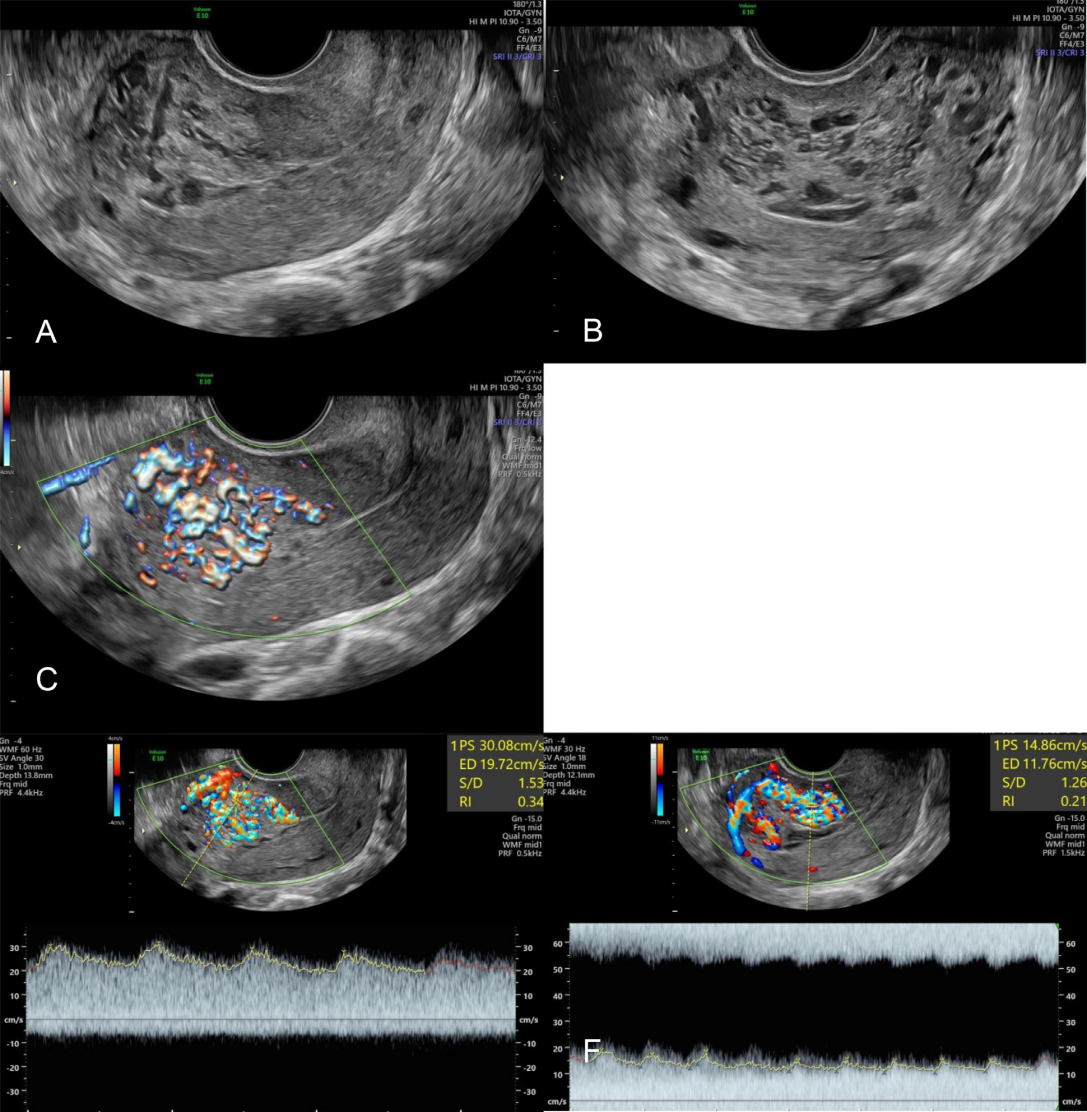
From top left showing heterogeneous tissue between the endometrium in (A) longitudinal view and (B) transverse view. (C and D) with color Doppler showing vascularity in the anterior wall of the uterus in longitudinal and transverse views, and lastly (E) and (F) showing Doppler flow with resistance indices (RI) of 0.34 and 0.21.

The following day the patient arrives to hospital by ambulance with continued severe vaginal bleeding, and is admitted to the ward with a hemoglobin of 72 g/L. Computerized tomography and also magnetic imaging showed a highly vascularized, expansive growth in the ventral part of the uterus, measuring 25 × 25 × 30 mm, containing several prominent and coiled arteries. The patient undergoes a second opinion ultrasound at a specialized ultrasound clinic in Lund, which concludes a diagnosis of classic uterine AVM Beta Hcg is measured at <1, hence ruling out hormone producing trophoblastic tumor (see Fig. 1). Dilatation and curettage was not recommended and only hysterectomy was to be considered if vitally indicated. The Department of vascular surgery was also consulted, and embolization was not recommended as the AVM was considered to have feeding arteries bilaterally from the uterine arteries, risking uterus necrosis.

The patient was planned for a vaginal hysterectomy the following day. During the night the patient, once again bleeds profusely, with approximately 1000 mL of acute blood loss. An emergency vaginal hysterectomy was performed during the night.

The final pathologic evaluation described a sharply distinguished tumor on the anterior wall of the uterus, which microscopically showed trophoblastic invasion deep down into the myometrium, surrounded by vessels of varied caliber and was considered to be the placental attachment existing abnormal blood vessel causing placenta increta. The consideration was made that this deep attachment was a remnant after a placenta increta, or conversely a pre-existing abnormal blood vessel causing placenta increta together with an atypical and coexistent placental site nodule.

## Discussion

We demonstrate a case of acute vaginal bleeding due to a suspected acquired uterine AVM, which led to an emergency hysterectomy despite repeated attempts at conservative management. The subsequent pathological examination revealed 3 findings, namely placenta increta, abnormal blood vessels and a placental site nodule prompting one to consider the connection between these entities.

In their study on assessing the spontaneous outcome of uterine vascular malformations detected with ultrasonography and color Doppler, Timmerman et al. [3] discovered no vascular malformations without prior pregnancy. They suggested that most cases of suspected acquired AVMs are in fact subinvolution of the placental bed and that true AVMs are exceedingly rare. Less than 100 cases are reported in the literature and it is hence difficult to establish the incidence of true AVMs [2].

Furthermore, Barber et al. [4] describes 3 case reports of identified AVMs following conservative management of placenta percreta, where AVM may represent the natural result from the erosive nature of the syncytiotrophoblastic tissue and chorionic villi to develop, establish and maintain blood supply of the placenta. In a further case report, Guan et al. [5] reports a case with an acquired uterine AVM due to a previous cornual pregnancy with placenta accreta, likely due to previous failed tubal ligation. Subsequently, Roach et al. [6] present the case of a postpartum patient with both a uterine AVM and retained placenta increta, suggesting the 2 complications may be related.

In a similar manner, placental site nodules (PSN) are defined as benign non-neoplastic remnants from a previous pregnancy and differ from placental trophoblastic disease which is considered malignant [7,8]. PSNs are thought to arise from intermediate trophoblasts, and to be caused by endometrial alterations leading to abnormal involution of the placental site [8]. Previous surgical interventions such as caesarean section and dilatation and curettage are believed to increase the risk of endometrial alteration, which may lead to defective implantation [8].

In our case, the findings of a blood vessel malformation with a remnant of placenta increta and a placental site nodule after a medical termination, give further evidence to the hypothesis of Timmerman et al. [3] that these conditions are related to subinvolution of the placental bed. In fact, it gives rise to the potential that PSN and blood vessel malformation are entities within the same spectrum in the pathological process of invasive placental disorders and that the vessel malformation that were assessed as AVM on ultrasound actually represents invasive placental disorder. Syncytioblasts and intermediate trophoblasts with their ability to erode and invade, and hence establish blood flow, could be considered to play a major role in the pathophysiology. We conclude that in the setting of a previous trophoblastic process within the uterus, the development of an AVM is to be considered part of abnormal placental invasion, rather than an entity of its own.
